# Antiferromagnetic Kondo lattice compound CePt_3_P

**DOI:** 10.1038/srep41853

**Published:** 2017-02-03

**Authors:** Jian Chen, Zhen Wang, Shiyi Zheng, Chunmu Feng, Jianhui Dai, Zhu’an Xu

**Affiliations:** 1 Department of Physics and State Key Laboratory of Silicon Materials, Zhejiang University, Hangzhou 310027, China; 2 Zhejiang University of Water Resources and Electric Power, Hangzhou 310018, China; 3 Department of Physics, Hangzhou Normal University, Hangzhou 310036, China; 4 Zhejiang California International NanoSystems Institute, Zhejiang University, Hangzhou 310058, China; 5 Collaborative Innovation Center of Advanced Microstructures, Nanjing 210093, China

## Abstract

A new ternary platinum phosphide CePt_3_P was synthesized and characterized by means of magnetic, thermodynamic and transport measurements. The compound crystallizes in an antiperovskite tetragonal structure similar to that in the canonical family of platinum-based superconductors *A*Pt_3_P (*A* = Sr, Ca, La) and closely related to the noncentrosymmetric heavy fermion superconductor CePt_3_Si. In contrast to all the superconducting counterparts, however, no superconductivity is observed in CePt_3_P down to 0.5 K. Instead, CePt_3_P displays a coexistence of antiferromagnetic ordering, Kondo effect and crystalline electric field effect. A field-induced spin-flop transition is observed below the magnetic ordering temperature *T*_*N*1_ of 3.0 K while the Kondo temperature is of similar magnitude as *T*_*N*1_. The obtained Sommerfeld coefficient of electronic specific heat is *γ*_*Ce*_ = 86 mJ/mol·K^2^ indicating that CePt_3_P is a moderately correlated antiferromagnetic Kondo lattice compound.

The interplay among spin, charge and orbital degrees of freedom in transition metal compounds has triggered enormous research interests in condensed matter physics and material science. For a large family of layered 3*d* electron superconductors (SCs) such as the copper oxides^1^ and iron pnictides^2^, the spin fluctuations caused by strong 3*d* electron correlations play a vital role in the unconventional superconductivity. Besides these 3*d* transition metal systems, several platinum-based SCs exhibit remarkably rich physical properties and therefore have also attracted considerable attention, partly owing to the moderately strong spin-orbit coupling of the platinum 5*d* electrons. The most prominent example is the heavy fermion noncentrosymmetric (NCS) SC CePt_3_Si, in which exotic superconductivity is observed below *T*_*c*_ = 0.75 K^3^: an admixture of spin-singlet and spin-triplet pairing symmetry, nodal gap structure and huge upper critical field (*B*_*c*2_ ≈ 4 T)^4^. The delicate interplay between the cerium 4*f* and the platinum 5*d* electrons places this material on the border of the magnetic quantum critical point (QCP) but still in the antiferromagnetic (AFM) gound state, rendering the role of inversion symmetry unclear^5^. Among a series of filled skutterudite *MT*_4_*X*_12_ (*M* = rare-earth or alkaline-earth metals, *T* = transition metals and *X* = P, As, Sb and Ge) with the cubic space group 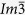 (No. 204), PrPt_4_Ge_12_ was reported to exhibit time-reversal symmetry breaking from zero-field *μ*SR measurements^6^. As a result of the unexpectedly high transition temperature *T*_*c*_ = 7.9 K and the moderately enhanced Sommerfeld coefficient *γ* = 76 mJ/mol ⋅ K^2^, PrPt_4_Ge_12_ has been extensively studied and multiband superconductivity has been proposed based on the analysis of the photoemission spectroscopy^7^ as well as the magnetic penetration depth^8^. Moreover, SrPtAs is recently reported to crystallize in a hexagonal structure (*P*6_3_/*mmc*, No. 194) with weakly coupled PtAs layers forming a honeycomb lattice^9^. The peculiar locally NCS structure within PtAs layer together with a strong spin-orbit coupling demonstrates SrPtAs as an attractive material to explore superconductivity with a spontaneous static magnetic field *B*_*s*_^10^.

It is interesting that among the platinum-based superconductors, the newly reported family of *A*Pt_3_P (*A* = Ca, Sr and La) shares the structural similarity with that of iron pnictides^11^. These compounds crystallize in a tetragonal structure with space group *P*4/*nmm* (No. 129) with stacking in the order of *A*-Pt_6_P-*A* along the *c*-axis. The distorted antiperovskite Pt_6_P octahedral unit alternates within the *ab* plane, forming an antipolar pattern. The *z* → −*z* inversion operation is thus preserved. Due to the structural distortion, the platinum atoms take two different sites as Pt(I) and Pt(II) so that the Pt(II) and P atoms form a Pt_2_P layer resembling the FeAs layer in the iron-based superconductors. Of course, the structure of *A*Pt_3_P is also somewhat similar to that of CePt_3_Si, but the latter is actually isotypic to the NCS compound CePt_3_B with the space group *P*4*mm* (No. 99)^3^. The corresponding Pt_6_Si unit has the polar structure under this space group leading to the absence of inversion symmetry, different from the antipolar structure in *A*Pt_3_P. Noticeably, the *A*Pt_3_P family shows a significant variation of *T*_*c*_, i.e., *T*_*c*_ = 8.4 K, 6.6 K and 1.5 K for *A* = Sr, Ca and La, respectively. It was reported theoretically that spin-orbit coupling (SOC) effect is significant in LaPt_3_P but negligible in CaPt_3_P and SrPt_3_P^12^,^13^,^14^. The origin of significantly enhanced *T*_*c*_ in SrPt_3_P is still debatable. It was suggested to be due to a possible dynamic charge-density-wave (CDW)^12^. However, a theoretical work by Zocco *et al*. indicated SOC could strongly renormalize the electron-phonon coupling of SrPt_3_P and thus enhance the electronic density of states near the Fermi level^15^. Moreover, several theoretical works claimed that the CDW instability could not be reproduced in SrPt_3_P^13^,^14^. The centrosymmetric (CS) compounds *A*Pt_3_P reported so far do not involve the 4*f* electrons. The interplay between strong 4*f* electron correlation and superconductivity of 5*d* electrons in the *A*Pt_3_P family remains an open issue.

In this paper, we report our successful synthesis of such a candidate compound CePt_3_P in the platinum-based phosphides *A*Pt_3_P family. We performed systematic measurements of the physical properties including the magnetic susceptibility, magnetization, specific heat and electrical resistivity. However, no evidence of superconductivity is observed down to 0.5 K in CePt_3_P, in contrast to other *A*Pt_3_P compounds. Instead, the compound displays the rich physics involving the coexistence of magnetic ordering, Kondo coherence as well as crystalline electric field (CEF) effect. We shall discuss these properties and highlight the delicate 4*f*-5*d* interplay in this system.

## Results and Discussion

**Figure 1:**
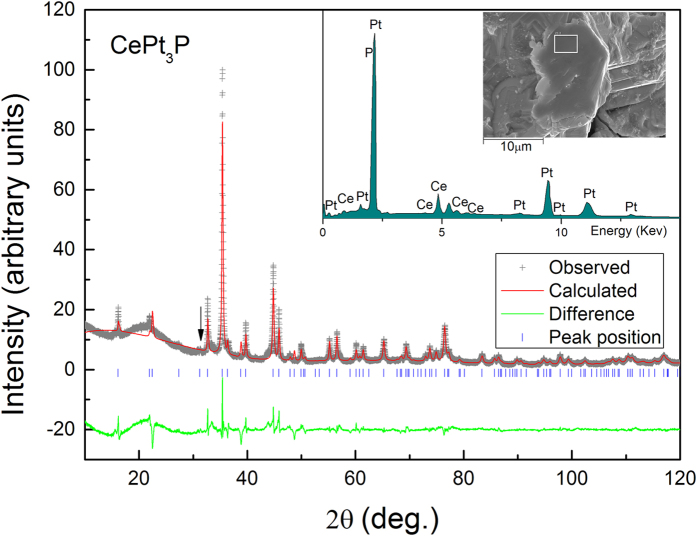
Rietveld refinement of the polycrystalline CePt_3_P XRD pattern. Arrow marks an impurity phase which might be PtP_2_. Inset shows a typical energy-dispersive x-ray spectrum with electron beams focused on the selected area of the as-grown sample.

Figure 1 shows the Rietveld refinement of the XRD pattern of polycrystalline CePt_3_P samples. Almost all peaks can be well indexed with the tetragonal structure with the space group *P*4/*nmm* (No. 129), except for a tiny peak of an impurity phase around 31.4° which might be PtP_2_. The result of the Rietveld refinement^16^ shows a good convergence: *R*_*wp*_ = 13.4%, *S* = 3.3. The refined lattice parameters of CePt_3_P are *a* = 5.7123(7) Å and *c* = 5.4679(6) Å as listed in Table 1. The room temperature XRD patterns of LaPt_3_P are also refined with *R*_*wp*_ = 14.9%, *S* = 2.7 (data not shown). The refined lattice parameters of LaPt_3_P are *a* = 5.7597(3) Å and *c* = 5.4736(3) Å. For comparison, the lattice parameters of the other *A*Pt_3_P compounds are also provided in Table 1. One can see obviously that *a* of CePt_3_P is smaller, while *c* is larger, compared with the lattice parameters of SrPt_3_P. Due to the lanthanide contraction, both of *a* and *c* of CePt_3_P are smaller than those of LaPt_3_P. From the EDS measurements, the molar ratio is Ce:Pt:P = (1.0 ± 0.1):(3.2 ± 0.2):(0.7 ± 0.2) for CePt_3_P and La:Pt:P = (1.0 ± 0.1):(2.6 ± 0.1): (0.8 ± 0.1) for LaPt_3_P. The actual chemical compositions are close to the nominal ones, while there seems a deficiency on the P site for both CePt_3_P and LaPt_3_P.

**Figure 2:**
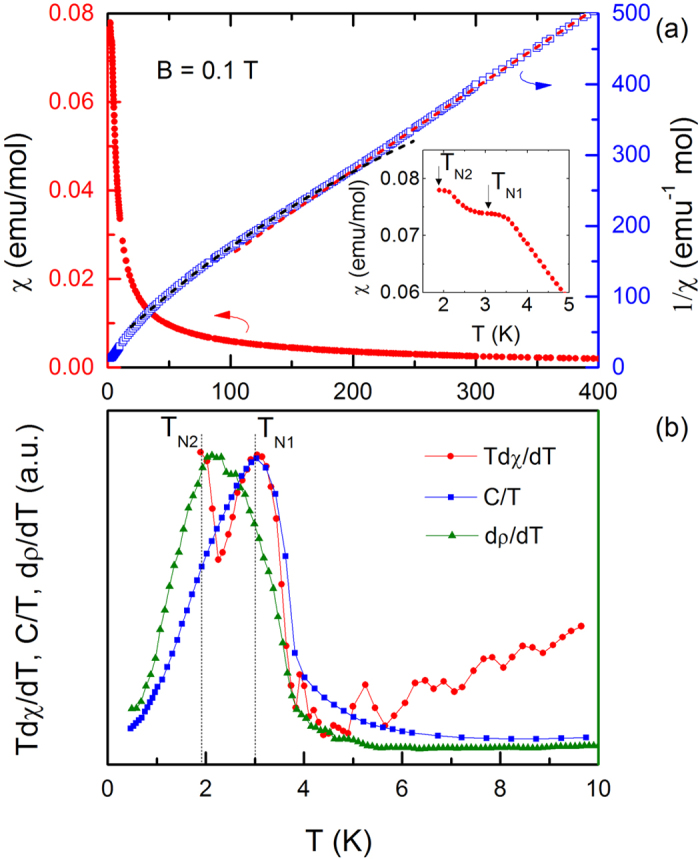
(**a**) Temperature dependence of magnetic susceptibility, *χ*, and inverse magnetic susceptibility, 1/*χ*, of CePt_3_P measured under magnetic field *B* = 0.1 T on the left and right axis, respectively. Two dashed lines show the Curie-Weiss fit for *T* > 200 K and *T* < 100 K, respectively. Inset: enlarged plot of *χ* at *T* < 5 K. (**b**) The AFM transition temperature *T*_*N*1_ and *T*_*N*2_ determined from the derivative susceptibility *T*d*χ*/d*T*, specific heat *C(T*)/*T* and derivative resistivity d*ρ*/d*T* (The complete data of specific heat and resistivity will be shown in the following figures).

The temperature-dependent molar magnetic susceptibility *χ(T*) = *M/H* and inverse magnetic susceptibility 1/*χ(T*) of CePt_3_P measured at *H* = 1000 Oe are presented in Fig. 2(a). *χ(T*) obeys a modified Curie-Weiss law above 200 K, *χ* = *χ*_0_ + 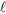/(*T* − *θ*). *χ*_0_ is a temperature independent susceptibility from the core diamagnetism, the van Vleck and Pauli paramagnetism, 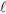 is the Curie constant and *θ* is the Weiss temperature. The relatively large absolute value of *θ* = −28.3 K may be attributed to the hybridization of the 4*f* electronic states with the conduction band^17^. The derived effective moment *μ*_*eff*_ = 2.52*μ*_*B*_ is almost equal to that of a free Ce^3+^ ion, indicating the trivalent Ce ion and well localized moment of Ce-4*f*^ 1^ electrons at high temperature. *χ*_0_ is in the magnitude order of 10^−3^. For *T* < 100 K, a change of the slope of 1/*χ(T*) can be clearly observed and the fitting parameters are *μ*_*eff*_ = 2.11*μ*_*B*_, and *θ* = −15.3 K. Here the change of the slope and the decreased value of *μ*_*eff*_ can be ascribed to the CEF effect. With decreasing temperature, *χ(T*) increases and shows a round peak around 3.0 K. Upon further cooling, another anomaly is observed near our base temperature. Two magnetic transition temperatures are determined from the peaks of derivative susceptibility *T*d*χ*/d*T* as *T*_*N*1_ = 3.0 K and *T*_*N*2_ = 1.9 K (seen from Fig. 2(b)). Considering the negative Weiss temperature, the first anomaly marks the AFM ordering below *T*_*N*1_ which is compatible with the magnetization measurement (discussed below). While the second anomaly is attributed to a spin-reorientation. A similar phenomenon was observed in CeNiAsO^18^. Further experimental studies, especially neutron diffraction measurement on single crystals of CePt_3_P, are necessary to clarify the magnetic structure at low temperature.

**Figure 3:**
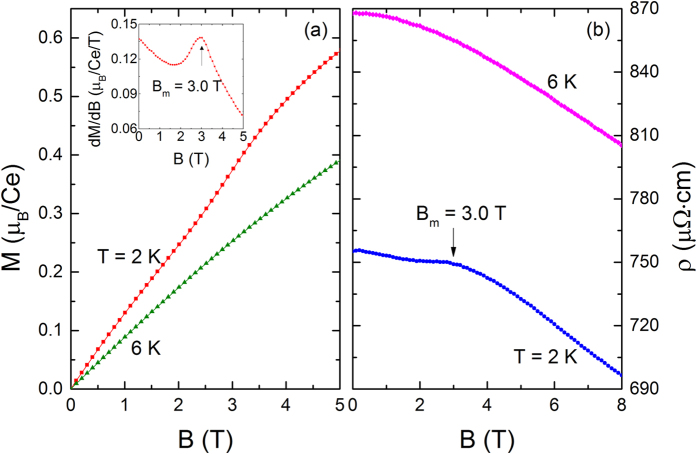
(**a**) Field dependence of the magnetization *M(B*). (**b**) Resistivity *ρ* of CePt_3_P vs. *B* measured at *T* = 2 and 6 K. Inset to (**a**) displays the derivative of the magnetization with respect to the field *dM/dB* for *T* = 2 K.

The isothermal magnetization *M(B*) of CePt_3_P, measured in the *B*-sweep mode containing both field-up and down loops, is displayed in Fig. 3(a). In the AFM ordering state, *M(B*) displays a linear field dependence when *B* < 2.0 T, but undergoes a weak step-like increase around 3.0 T. This anomaly, which is ascribed to a field-induced metamagnetic transition (MMT), can be independently determined to be *B*_*m*_ = 3.0 T by the peak in *dM/dB* curve (inset to Fig. 3(a)) and the hump in *ρ(B*) curve (Fig. 3(b)) measured at *T* = 2 K. The expected hysteresis around *B*_*m*_ is not observed and such absence of hysteresis around MMT was also reported in the single-crystalline samples CeAuSb_2_^19^ and YbNiSi_3_^20^. No hysteresis in resistivity is observed for CePt_3_P in this magnetic field range either. Note that the *M(B*) curve does not show a saturation trend in the highest field limit and the value *M* ~ 0.6*μ*_*B*_ at *B* = 5 T is much lower than the theoretical value of 2.14*μ*_*B*_ for the saturated moment of free Ce^3+^ ions which is probably due to the CEF effect. Figure 3(b) shows the isothermal resistivity versus the applied field. *ρ* decreases monotonously with increasing magnetic field at *T* = 6 K > *T*_*N*1_. Whereas at *T* = 2 K < *T*_*N*1_, a hump around *B*_*m*_ = 3.0 T is added to the decreasing trend. This feature is compatible with the MMT observed in the magnetization measurement.

**Figure 4:**
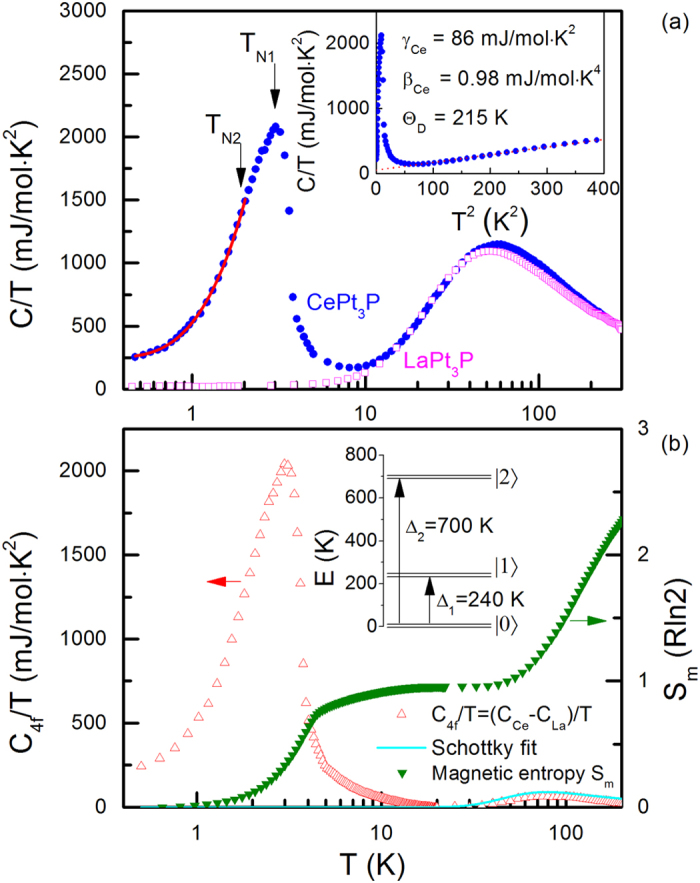
(**a**) Specific heat divided by temperature, *C/T*, versus log*T*. The solid symbols are for CePt_3_P, while the open symbols represent the non-magnetic compound LaPt_3_P. The solid line is a fit to Eq. 5 for *T* ≤ 1.9 K. (**b**) The Ce-4*f* contribution, *C*_4*f*_/*T*, and the magnetic entropy, *S*_*m*_, on the left and right axis, respectively, measured at zero magnetic field plotted in a logarithmic temperature scale for *T* = 0.4–200 K. The solid line shows the Schottky anomaly contribution *C*_*Sch*_. Inset to (**a**) shows *C/T* versus *T*^2^ together with the fitting parameters for CePt_3_P (see the text): the Sommerfeld coefficient *γ*_*Ce*_, *β*_*Ce*_ and the Debye temperature Θ_*D*_. The dashed line is a linear fit in the temperature range *T* = 10–20 K. Inset to (**b**) displays the schematic sketch of CEF energy levels for Ce^3+^ ion in CePt_3_P.

The specific heats of CePt_3_P and LaPt_3_P divided by *T, C(T*)/*T*, are plotted in the main panel of Fig. 4(a) in a semi-logarithm scale. At room temperature, *C(T*) saturates to about 135 and 140 J/mol ⋅ K for La and Ce compound, respectively, which are, within an acceptable error range, compatible with the classical Dulong-Petit law 3*NR* with *N* = 5 and *R* = 8.31 J/mol ⋅ K, where *R* is the universal gas constant. The specific heat *C(T*) of LaPt_3_P is typical for nonmagnetic metals since no typical anomaly can be observed at high temperature. At low temperature, the specific heat of LaPt_3_P is dominated by the electronic and phonon contributions for *T* < Θ_*D*_/10, therefore, it can be fitted to a power law *C/T* = *γ*_*La*_ + *β*_*La*_*T*^2^ over 10–20 K (data not shown). Here Θ_*D*_ is the Debye temperature, and *γ*_*La*_ and *β*_*La*_ denote the coefficients of the electronic and phonon contributions, respectively. It should be noted that there is a small jump around 1 K in the specific heat of LaPt_3_P which should correspond to a superconducting transition though it is too small to observe in Fig. 4.

In the paramagnetic region above the magnetic transition, the specific heat of CePt_3_P can be expressed as


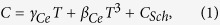


where the coefficients *γ*_*Ce*_ and *β*_*Ce*_ are of electronic and phonon contributions of CePt_3_P, respectively, while *C*_*Sch*_ describes the Schottky anomaly item. A linear *T*^2^-dependence is clearly seen in *C/T* vs *T*^2^ plot for temperature below 20 K (see inset to Fig. 4(a)). The derived Sommerfeld coefficient is *γ*_*Ce*_ = (86 ± 1) mJ/mol ⋅ K^2^. The value is moderately enhanced by a factor of 57 compared with that of LaPt_3_P where *γ*_*La*_ = (1.5 ± 0.1) mJ/mol ⋅ K^2^, manifesting the correlation effect contributed from the Ce-4*f* electrons. Therefore, CePt_3_P is a Kondo lattice compound due to the strong 4*f* electron correlation and moderate effective 4*f*−5*d* hybridization. Note that *γ*_*La*_ for LaPt_3_P derived here is slightly smaller but still in the same magnitude order with that obtained in ref. 11. The reported phonon coefficients are in reasonable agreement with each other: *β*_*Ce*_ = 0.98(1) mJ/mol ⋅ K^4^ for CePt_3_P and *β*_*La*_ = 0.94(1) mJ/mol ⋅ K^4^ for LaPt_3_P, indicating similar phonon contributions. The Debye temperature Θ_*D*_ estimated by using Θ_*D*_ = (12*π*^4^*NR*/5*β*)^1/3^ is (215 ± 1) K for CePt_3_P and (218 ± 1) K for LaPt_3_P, implying that the above analysis is quite self-consistent.

The Ce-4*f* contribution to the specific heat of CePt_3_P is then deduced by subtracting the measured specific heat of the nonmagnetic isostructural reference sample LaPt_3_P from the total specific heat of CePt_3_P, i.e., *C*_4*f*_ = *C*_*Ce*_ − *C*_*La*_. The result is shown in the main panel of Fig. 4(b), plotted as *C*_4*f*_/*T* vs *T* in a logarithmic scale. The Schottky anomaly, which is visible as a broad peak centered around 90 K in *C*_4*f*_/*T* curve, should be caused by the excitations between different CEF levels. The Schottky anomaly with three Kramers doublets (one doublet ground state and two excited doublets) for Ce^3+^ ion with *j* = 5/2 experiencing a tetragonal crystal-field potential can be expressed by refs 21,22


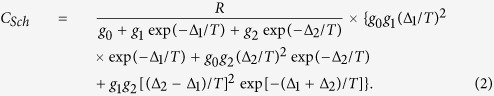


Here *g*_*i*_ = 2 is the degeneracy of the *i*th doublet state and Δ_*i*_ is the energy difference between the ground state and the *i*-th excited state (see the schematic sketch drawn in the inset of Fig. 4(b)). Eq. 2 is applied to *C*_4*f*_/*T* of CePt_3_P over a temperature range of 50–130 K. The derived CEF energy differences are Δ_1_ = (20.9 ± 0.1) meV (~(240 ± 1) K) and Δ_2_ = (60.9 ± 0.3) meV (~(700 ± 3) K). This result may explain the slope change in 1/*χ(T*) curve as well as the broad hump in both *ρ*_*mag*_ and *S*. Furthermore, the large value of Δ_1_ is consistent with the reduced effective Ce moment below 100 K. The magnetic entropy gain *S*_*m*_ is calculated by integrating *C*_4*f*_/*T* over *T* and plotted on the right axis in Fig. 4(b). One can see that *S*_*m*_ reaches about 0.51*R*ln2 at *T*_*N*1_ and *R*ln2 is recovered at ~50 K, indicating that the ground state with the AFM ordering of Ce^3+^ moments is Kramers two-fold degenerate. The plateau over the temperature range of *T* = 10–30 K indicates that the first excited CEF level is far above *T*_*N*1_. *S*_*m*_ reaches *R*ln4 at ~150 K and increases substantially above the Schottky anomaly. For a Kondo lattice, the Kondo temperature can be estimated by the magnetic entropy at *T*_*N*_ via ref. 23


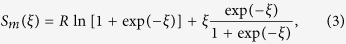


where *ξ* = *T*_*K*_/*T*_*N*_. The yielded *T*_*K*_ is about (6.1 ± 0.1) K for CePt_3_P.

At low temperature, *C*_4*f*_/*T* shows a pronounced *λ*-shape peak at *T*_*N*1_ = 3.0 K, implying a second-order phase transition. The expected jump in specific heat is 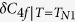 ~6 J/mol ⋅ K. A slight slope change in *C*_4*f*_/*T* is also observed around *T*_*N*2_ = 1.9 K, consistent with the low-temperature anomaly observed in aforepresented *χ(T*) curve. Based on the mean-field theory of Besnus *et al*.^24^ and Bredl *et al*.^25^, the specific heat jump 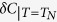 is related to the Kondo temperature *T*_*K*_ by the following formula


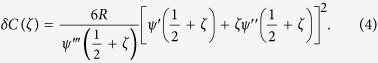


Here *ζ* = (*T*_*K*_/*T*_*N*_)/2*π, ψ* denotes the digamma function and *ψ*′, *ψ*′′ and *ψ*′′′ are the first three derivatives of *ψ*. Then the Kondo temperature can be also estimated by applying Eq. 4, obtaining a ratio of *T*_*K*_/*T*_*N*1_ = 0.88, or *T*_*K*_ ~ (2.7 ± 0.1) K. Therefore, based on both magnetic entropy and specific heat jump, it is reasonable to estimate *T*_*K*_ ~ 2–6 K in this compound.

In the magnetically ordered state, the AFM spin-wave spectrum follows a dispersion relation of *ε*_*k*_ =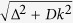. Here *ε*_*k*_ is the excitation energy, Δ is the gap in the spin-wave spectrum, and *D* is the spin-wave stiffness. The phonon contribution, *β*_*Ce*_*T*^3^ item, can be subtracted from the total specific heat *C* as Δ*C* = *C* − *β*_*Ce*_*T*^3^. At low temperature, Δ*C* is described by the following expression^26^,^27^:


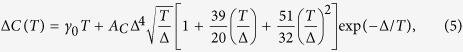


where the coefficient *A*_*C*_ is proportional to *D*^−3^. Fitting the specific heat below *T*_*N*2_ (solid line in Fig. 4(a)) gives the fitting parameters *γ*_0_ = 247 mJ/mol ⋅ K^2^, Δ = 2.6 K, and *A*_*C*_ = 67.5 mJ/mol ⋅ K^4^. The considerably enhanced zero-temperature Sommerfeld coefficient *γ*_0_ is about 3 times of *γ*_*Ce*_ obtained in the paramagnetic state, indicating the formation of moderate-heavy quasiparticles in the antiferromagnetically ordered state. It is worthwile noting that the obtained spin-wave gap Δ is of the order of magnitude often found in cerium intermetallics with AFM ground states^17^.

**Figure 5:**
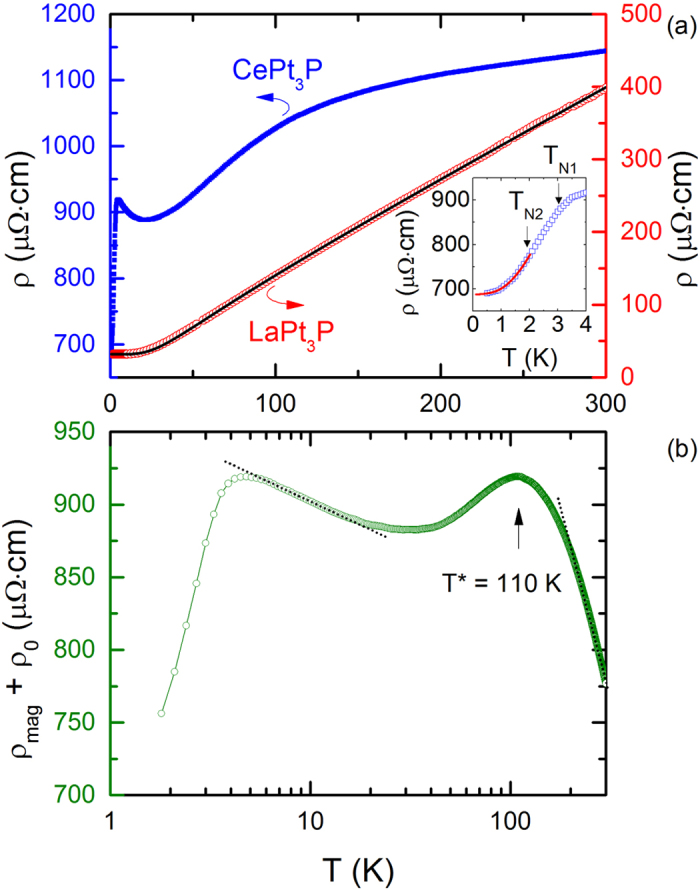
Transport properties as a function of temperature. (**a**) *ρ(T*) of CePt_3_P and LaPt_3_P in a linear temperature scale. The solid line is a fit to the Bloch-Grüneissen-Mott formula (Eq. 6). (**b**) The magnetic contribution to the electrical resistivity of CePt_3_P, *ρ*_*mag*_, versus log*T*. The dashed lines display linear fits in the low and the high temperature regions, respectively. Inset to (**a**) plots a fit to Eq. 8 below *T* ≤ 1.9 K.

The temperature variation of the electrical resistivity of CePt_3_P, *ρ(T*), is plotted in Fig. 5(a). The resistivity at room temperature is *ρ*_300*K*_ = 1140 *μ*Ω ⋅ cm, a value rather typical for the Ce-based Kondo compounds with narrow *f*-band^28^. The resistivity decreases with decreasing temperature and exhibits two features. A broad hump around 110 K reflects the 4*f*-electron contribution via Kondo scattering from different CEF levels^21^,^22^. At low temperature, a pronounced peak in *ρ(T*) around 3 K is directly visible, indicating the AFM ordering phase below *T*_*N*1_ = 3.0 K. Above *T*_*N*1_, *ρ* increases in a minus logarithmic temperature manner over *T* = 5–20 K, reflecting the Kondo-type scattering. Further evaluation of *ρ(T*) requires information of the phonon contribution which could be taken from the homologous and isostructural analog, LaPt_3_P. The *ρ(T*) of LaPt_3_P, which is also presented in Fig. 5(a), can be well described by a Bloch-Grüeneisen-Mott (BGM) relation:


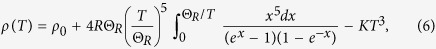


where *ρ*_0_ is the residual resistivity due to lattice defects, the second term denotes electron-phonon scattering, and the third one accounts for the contribution due to Mott’s *s*-*d* interband electron scattering. A least square fitting of the BGM formula to the experimental data over the temperature range 2–300 K leads to the following parameters: *ρ*_0_ = 32 *μ*Ω ⋅ m, Θ_*R*_ = 160 K, *R* = 1.25 *μ*Ω ⋅ cm/K, and *K* = 4.1 × 10^−8^ *μ*Ω ⋅ cm/K^3^. Note that the residual resistivity *ρ*_0_ is smaller than that in ref. 11. The parameter Θ_*R*_ is usually considered as an approximation of the Debye temperature Θ_*D*_ in spite of some contribution due to electron-electron correlations in Θ_*R*_^29^. Θ_*D*_ yielded from the specific heat data is 218 K which is in accordance with Θ_*R*_ from the resistivity data. LaPt_3_P exhibits simple metallic behavior as we expected, without the characteristic features due to the interplay of Kondo and CEF effects in CePt_3_P mentioned above.

In order to analyze the magnetic contribution to the electrical resistivity of CePt_3_P, it is reasonable to assume that the phonon contribution in this compound can be properly approximated by that in LaPt_3_P, *ρ*_*ph*_ = *ρ(La*) − *ρ*_0_(*La*), so we have


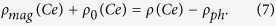


The temperature dependence of *ρ*_*mag*_ + *ρ*_0_ derived in this way is presented in Fig. 5(b) in a semilogarithmic scale. As a distinct feature in a Kondo lattice system, a pronounced broad hump centered at T^*^ = 110 K become obvious in *ρ*_*mag*_ curve, which could be ascribed to the Kondo scattering from different CEF levels. According to Cornut and Coqblin^21^, this maximum provides an estimate of the CEF splitting energy scale ~200 K of Ce-4*f*^1^ state with *j* = 5/2. On the other hand, as temperature is decreased, *ρ*_*mag*_ increases in a logarithmic scale, as shown as the dotted lines in Fig. 5(b) above *T* > 200 K and between 5–20 K, respectively. Following the theoretical predictions of Cornut and Coqblin^21^, the logarithmic slopes 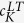 and 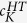 in the low-temperature and high-temperature regions, respectively, are proportional to the squared effective degeneracy *λ* of the thermally populated levels: *c*_*K*_ ∝ *λ*^2^ − 1. For cerium compouns with Ce^3+^ ion placed in a noncubic crystalline environment the ground multiplet splits into three doublets, thus the expected ratio is 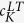 : 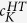 = 3:35. In the case of CePt_3_P, with the coefficients 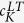 = −0.063 and 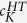 = −0.57 yielded from linear fitting of *ρ*_*mag*_ vs log*T* (see the dashed lines in Fig. 5(b)), the ratio is about 3:27, reasonably close to the theoretical prediction.

From the inset of Fig. 5(a), *ρ* drops rapidly below about 3.0 K owing to the reduction of spin-flip scattering upon entering the AFM ordered state. This magnetic transition temperature is determined from a slope change of d*ρ*/d*T* in Fig. 2(b). Upon further cooling, a second slope change in *ρ* is observed around 1.9 K, corresponding to the pronounced kink in d*ρ*/d*T*. Therefore, two magnetic transitions in CePt_3_P are apparent from the analysis of magnetic susceptibility *χ(T*), specific heat *C(T*) and electrical resistivity *ρ(T*), as shown in Fig. 2(b): the first transition *T*_*N*1_ corresponds to the AFM ordering temperature, while the second one *T*_*N*2_ is presumably associated with the spin reorientation. The values of *T*_*N*1_ and *T*_*N*2_ derived from different measurements agree well with each other. It is noted that while LaPt_3_P shows superconductivity around *T*_*c*_ = 1.0 K (from specific heat), no superconductivity is observed in CePt_3_P down to 0.5 K.

Considering the relativistic dispersion relation for the AFM magnon spectrum, the electrical resistivity *ρ(T*) for *T* < Δ can be well described by the following equations^26^,^27^:


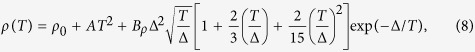


where *ρ*_0_ is the temperature-independent residual resistivity, the constant coefficient *B*_*ρ*_ is related to the spin-wave stiffness *D* by the proportionality *D*^−3/2^ and Δ is the same gap in the spin-wave spectrum as in Eq. (5). *AT*^2^ stems from the electron-electron scattering following the Fermi liquid theory, while the third term describes the electron-magnon scattering. This formula is applied to the electrical resistivity of CePt_3_P (dotted line in the inset of Fig. 5(a)) and a very good fit is obtained with the fitting parameters: *ρ*_0_ = 688 *μ*Ω ⋅ cm, Δ = 4.0 K, *A* = 9.0 *μ*Ω ⋅ cm/K^2^ and *B*_*ρ*_ = 25 *μ*Ω ⋅ cm/K^2^. Considering the relatively short fitting range of temperature, the derived Δ value for the measured polycrystalline sample is still reasonably compared with that obtained from the specific heat data.

Based on the above analyses, CePt_3_P displays the coexistence of three important characteristics: AFM ordering of the cerium local moments due to the Ruderman-Kittel-Kasuya-Yosida exchange interaction, the Kondo effect due to the strong 4*f* electron correlation and moderate effective 4*f*−5*d* hybridization, and the CEF interactions. The AFM ordering at *T*_*N*1_ = 3.0 K is clearly identified by the pronounced anomalies in the temperature-dependent magnetic, thermodynamic and electrical measurements. In addition, another anomaly at *T*_*N*2_ = 1.9 K is also visible from the physical properties, and is probably due to a change in the magnetic configuration within the AFM ordered phase. The behavior of *ρ(T*) and *C(T*) in the ordered region is well describable in terms of AFM spin-wave spectrum. The field-dependent behavior of the magnetization and electrical resistivity also indicates a MMT from the magnetic ordering to a spin-polarized state around *B*_*m*_ = 3.0 T. The magnetic structure of CePt_3_P is still unclear and the neutron diffraction or Mössbauer spectroscopy experiments are helpful to clarify the details of the magnetic structure.

The Kondo effect displays itself by the large value of Weiss temperature *θ* (compared with the ordering temperature), the reduced magnetic entropy and the specific heat jump at *T*_*N*_, as well as the enhanced Sommerfeld coefficient *γ*_*Ce*_. From the analysis of the specific heat data, the Kondo temperature *T*_*K*_ is estimated to be in the range of 2–6 K. Its value can be also estimated from the magnetic susceptibility as *T*_*K*_ ~ |*θ*|/4 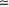 7.1 K^30^, in reasonable agreement with other estimates. Also, the Kondo effect is well manifested in the electrical resistivity for Kondo systems with strong CEF interactions which follows the negative logarithmic-temperature dependence as *ρ(T*) = *ρ*_0_ + 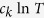, with Kondo coefficient *c*_*k*_ < 0^21^. The inverse susceptibility (1/*χ(T*)) curve shows a slope change between *T* = 100–200 K which is also attributed to the CEF effect. This temperature region is in accordance with the energy scale Δ_1_ = 240 K of the multiplet Ce^3+^ ion estimated from the Schottky contributions of the specific heat^21^,^22^.

Finally, it is very interesting to compare this CS compound CePt_3_P with the extensively studied NCS heavy fermion SC CePt_3_Si (*T*_*c*_ = 0.75 K)^3^. The crystal structure of CePt_3_P consists of alternative stacking of layers of Ce atoms and layers of distorted antiperovskite Pt_6_P octahedral units along the *c*-axis. The Pt_6_P octahedra is asymmetrically distorted perpendicular to the *ab*-plane but alternatively distributed in the *ab*-plane, resulting in a symmetric antipolar analogue of CePt_3_Si. CePt_3_Si shows antisymmetric spin-orbit coupling of the platinum 5*d* electrons due to the absence of *z* → −*z* symmetry as well as mixing spin-singlet and spin-triplet pairing states. The parity mixing alone can hardly account for the heavy fermion phenomena unless the strong electron-electron correlation effects which are ensured by the presence of Ce^3+^ ions are taken into consideration together^31^. Correspondingly, the suppression of superconductivity in CePt_3_P may be attributed to the enhanced AFM ordering. CePt_3_P is, therefore, probably placed further away from the magnetic QCP compared with CePt_3_Si (*T*_*N*_ = 2.2 K). With an external control parameter *δ*, such as doping or positive pressure, the system may be shifted towards *T*_*N*_ = 0, namely the QCP^32^,^33^. It is thus of great interest to investigate whether superconductivity exists in CePt_3_P at even lower temperature than 0.5 K; if superconductivity does exist, it will provide strong evidence for the proximity to a magnetic QCP in CePt_3_P. Comparing with CePt_3_P, the occurrence of superconductivity at *T*_*c*_ = 0.75 K in CePt_3_Si implies that the NCS crystal structure may favor unconventional superconductivity within the AFM ground state.

## Conclusion

In summary, we report the successful synthesis of a new compound CePt_3_P. From the collected experimental data of magnetization, specific heat and transport measurements, this compound is characterized as an antiferromagnetic Kondo lattice with crystal electric field effect. Two successive magnetic transitions of Ce 4*f* moments are observed: the magnetic ordering at *T*_*N*1_ = 3.0 K and the spin reorientation at *T*_*N*2_ = 1.9 K. Considering the moderately enhanced Sommerfeld coefficient of *γ*_*Ce*_ = 86 mJ/mol ⋅ K^2^ in the paramagnetic region and large value of *γ*_0_ = 247 mJ/mol ⋅ K^2^ in the the AFM region, the Kondo effect and the AFM order should coexist in the ground state. Thus a relatively large Fermi surface formed by the heavy quasiparticles is expected in CePt_3_P with a Kondo temperature *T*_*K*_ ~ 2–6 K. The *ab* initio crystal-field and electronic band structure calculations are necessary to further complement the present results. Further experiments such as chemical doping are presently underway in order to tune the ground state from the AFM ordering to strongly-correlated paramagnetic region.

## Experimental Methods

The polycrystalline sample of CePt_3_P was synthesized by solid state reaction. Ce piece (99.8%), Pt powder (99.9%) and P lump (99.999%) of high purity from Alfa Aesar were used as starting materials. Firstly, CeP was pre-synthesized by reacting Ce and P at 1173 K for 72 h. Secondly, powders of CeP and Pt were weighed according to the stoichiometric ratio, thoroughly ground and pressed into pellets. The pellets were then packed in Al_2_O_3_ crucibles and sealed in an evacuated quartz tube which were slowly heated to 1273 K and kept at that temperature for 7 days. Finally, the samples were thoroughly ground, cold pressed and annealed in vacuum to improve the sample homogeneity. For comparison, the polycrystalline sample LaPt_3_P was also synthesized in the similar process. All the preparation procedures except heating were carried out in an argon protected glove box with the water and oxygen content below 0.1 ppm. The obtained CePt_3_P sample is less compact than LaPt_3_P and both of them are quite stable in the air.

Powder x-ray diffraction (XRD) measurements at room temperature were carried out on a PANalytical x-ray diffractometer (Model EMPYREAN) with a monochromatic Cu *K*_*α*1_ radiation and a graphite monochromator. Lattice parameters were derived by Rietveld refinement using the program RIETAN 2000^16^. The energy dispersion x-ray spectroscopy (EDS) analysis was performed on a EDS spectrometer affiliated to a field emission scanning electron microscope (FEI Model SIRION). The electron beam was focused on a crystalline grain and the chemical compositions were averaged on at least 4 EDS spectra from different grains. The electrical resistivity *ρ(T*) was measured by the standard four-probe method in a Quantum Design physical property measurement system (PPMS-9). The dc magnetization was measured in a Quantum Design magnetic property measurement system (MPMS-5) with the temperature range of *T* = 2-400 K. The specific heat measurements were performed in the PPMS-9 down to about 0.5 K.

## Additional Information

**How to cite this article**: Chen, J. *et al*. Antiferromagnetic Kondo lattice compound CePt_3_P. *Sci. Rep.* **7**, 41853; doi: 10.1038/srep41853 (2017).

**Publisher's note:** Springer Nature remains neutral with regard to jurisdictional claims in published maps and institutional affiliations.

## Figures and Tables

**Table 1:** Comparisons of physical parameters among the *A*Pt_3_P family with *A* = Sr, Ca, La and Ce.

	SrPt3P	CaPt3P	LaPt3P	CePt_3_P
*a* (Å)	5.8094	5.6673	5.7597	5.7123
*c* (Å)	5.2822	5.4665	5.4736	5.4679
*z*_Pt(II)_	0.1409	—	0.1459	0.1582
*z*_P_	0.7226	—	0.7691	0.8310
*T*_*c*_/*T*_*N*_ (K)	8.4	6.6	1.5	3.0 (*T_N_*_1_)
				1.9 (*T_N_*_2_)
*ρ*_0_ (*μ*Ω ⋅ cm)	140	—	32	688
*γ*_0_ (mJ/mol ⋅ K^2^)	12.7	17.4	1.5	86

Atomic positions: *A* (0, 0, 0), Pt(I) (1/4, 1/4, 1/2), Pt(II) (0, 1/2, *z*_Pt(II)_), P (0, 1/2, *z*_P_). Note that data of Sr and Ca are taken from ref. 11.
